# Retraction: Mitostatin is down-regulated in human prostate cancer and suppresses the invasive phenotype of prostate cancer cells

**DOI:** 10.1371/journal.pone.0341382

**Published:** 2026-01-22

**Authors:** The PLOS One Editors

Following the publication of this article [1] concerns were raised with results presented in Figs 1-3. Specifically:

- There appears to be a vertical discontinuity between lanes 3 and 4 in the left β-actin panel in Fig 1A.
- When levels are adjusted to visualize the background, the bands in lane 3 in the left β-actin panel, and lane 2 in the right β-actin panel in Fig 1A appear to be discontinuous with adjacent regions of the image.
- In the left-most Mitostatin panel of Fig 1B, the bands in lanes 1 and 2 appear similar when flipped horizontally, although there appears to be additional signal below the left portion of the band in lane 2.
- There appears to be a vertical discontinuity between lanes 2 and 3 in the middle Mitostatin panel in Fig 1B.
- There are similarities between data representing different experiments reported in Fig 1B of [1] and Fig 6A of [2], specifically:
  - Mitostatin data in lanes 1, 3, 4 of the left panel of Fig 1B in [1] appear similar to Mitostatin data in lanes 1, 2, 3 of Fig 6A in [2].
  - Mitostatin data in the right panel of Fig 1B in [1] appear similar to Mitostatin data in lanes 4-8 of Fig 6A in [2].
  - The β-actin data in the left panel of Fig 1B in [1] appears similar to data in lanes 1-4 of the β-actin panel of Fig 6A in [2], when aspect ratio is adjusted.
  - β-actin data in the right panel of Fig 1B of [1] appear similar to data in lanes 4-8 of the β-actin panel in Fig 6A in [2], when aspect ratio is adjusted.
- In Fig 2, the images shown for PC3 V5 in panel A and for DU145 M2 in panel B appear similar when the DU145 M2 image is rotated 80°.
- In Fig 3B, the 8h PC3 panel appears to overlap with the 8h PC3 M2 panel.

The corresponding author stated that the panels included in [1] represent the original experimental outputs as obtained at the time of the study, with only uniform, global adjustments to brightness and contrast applied. They also stated that the underlying data for the above figures are no longer available. The corresponding author provided original plate images for Fig 2 which did not resolve the concerns.

In light of the above concerns which call into question the reliability and integrity of the published results, the *PLOS One* Editors retract this article.

DDA and RVI did not agree with the retraction. MF, AM, and RB did not agree with the retraction and stand by the article’s findings. JL, AV, MPG, PMC, BW, MR, DSB and LGG either did not respond directly or could not be reached.

The left and right Mitostatin and β-actin panels of Fig 1B report material similar to that published in Fig 6A in [2], published in 2008 by Springer Nature. The left and right Mitostatin and β-actin panels of Fig 1B are not offered under a CC BY license and are therefore excluded from this article’s [1] license.
